# Response inhibition deficits in externalizing child psychiatric disorders: An ERP-study with the Stop-task

**DOI:** 10.1186/1744-9081-1-22

**Published:** 2005-12-09

**Authors:** Björn Albrecht, Tobias Banaschewski, Daniel Brandeis, Hartmut Heinrich, Aribert Rothenberger

**Affiliations:** 1 Child and Adolescent Psychiatry, University of Göttingen, Germany; 2 Child and Adolescent Psychiatry, University of Zürich, Switzerland; 3 Child and Adolescent Psychiatry, University of Erlangen, Germany; 4 Heckscher-Klinik, München, Germany

**Keywords:** Event related potential, Stop task, Horse Race Model, response inhibition, attention deficit hyperactivity disorder, conduct disorder, comorbidity, children

## Abstract

**Background:**

Evidence from behavioural studies suggests that impaired motor response inhibition may be common to several externalizing child psychiatric disorders, although it has been proposed to be the core-deficit in AD/HD. Since similar overt behaviour may be accompanied by different covert brain activity, the aim of this study was to investigate both brain-electric-activity and performance measures in three groups of children with externalizing child psychiatric disorders and a group of normal controls.

**Methods:**

A Stop-task was used to measure specific aspects of response inhibition in 10 children with attention-deficit hyperactivity disorder (AD/HD), 8 children with oppositional defiant disorder/conduct disorder (ODD/CD), 11 children with comorbid AD/HD+ODD/CD and 11 normal controls. All children were between 8 and 14 years old. Event-related potentials and behavioural responses were recorded. An initial go-signal related microstate, a subsequent Stop-signal related N200, and performance measures were analyzed using ANCOVA with age as covariate.

**Results:**

Groups did not differ in accuracy or reaction time to the Go-stimuli. However, all clinical groups displayed reduced map strength in a microstate related to initial processing of the Go-stimulus compared to normal controls, whereas topography did not differ. Concerning motor response inhibition, the AD/HD-only and the ODD/CD-only groups displayed slower Stop-signal reaction times (SSRT) and Stop-failure reaction time compared to normal controls. In children with comorbid AD/HD+ODD/CD, Stop-failure reaction-time was longer than in controls, but their SSRT was not slowed. Moreover, SSRT in AD/HD+ODD/CD was faster than in AD/HD-only or ODD/CD-only. The AD/HD-only and ODD/CD-only groups displayed reduced Stop-N200 mean amplitude over right-frontal electrodes. This effect reached only a trend for comorbid AD/HD+ODD/CD.

**Conclusion:**

Following similar attenuations in initial processing of the Go-signal in all clinical groups compared to controls, distinct Stop-signal related deficits became evident in the clinical groups. Both children with AD/HD and ODD/CD showed deficits in behavioural response-inhibition accompanied by decreased central conflict signalling or inhibition processes. Neither behavioural nor neural markers of inhibitory deficits as found in AD/HD-only and ODD/CD-only were additive. Instead, children with comorbid AD/HD+ODD/CD showed similar or even less prominent inhibition deficits than the other clinical groups. Hence, the AD/HD+ODD/CD-group may represent a separate clinical entity.

## Background

Attention-deficit hyperactivity disorder (AD/HD) is characterised by symptoms of severe inattention, overactivity and impulsiveness. With its prevalence of 3–5% in school-age-children, AD/HD is one of the most common disorders in child and adolescent psychiatry [1]. According to Barkley's theory of AD/HD [2,3], deficient behavioural inhibition is the core deficit of the disorder, and may lead to impairments of executive functions. Behavioural inhibition may be separated into three interrelated processes called 'inhibition of the initial prepotent response to an event', 'stopping of an ongoing response' and 'interference control'.

Several behavioural studies reported deficits of response-inhibition in children with AD/HD ([4-8]; for a review see [9]). However, impaired behavioural response inhibition is also observed in children with other disruptive disorders such as ODD/CD [9], which is the most prevalent comorbidity of AD/HD and poses significant additional clinical and public health problems. In addition, further deficits which are not likely to result from deficient inhibition are present in children with AD/HD, as evident from their poor performance in a variety of executive functions tasks such as the Continuous Performance Test (CPT) [10,11], Wisconsin Card-Sorting-Task [12-14], Tower-of-Hanoi [13,14] and Stroop-Test [12,15]; for a review see [16,17].

In a more neurophysiologically oriented theory covering both ADHD and ODD/CD, Quay [18,19] following Gray [20] argued that the behavioural activation system (BAS, sensitive to reward) and the behavioural inhibition system (BIS, sensitive to punishment) may reflect distinct pathways for inhibition deficits. Children with AD/HD may suffer from an underactive BIS while their BAS seems to be unimpaired, whereas children with ODD/CD should have an overactive BAS that dominates their (unimpaired) BIS. Therefore, according to Quay's theory both AD/HD and ODD/CD groups should display deficits in inhibition, but for very different reasons. If comorbid AD/HD+ODD/CD is an additive combination of AD/HD and ODD/CD, this group should display the worst impairment in response inhibition because an overactive BAS may be combined with a weak BIS. Concerning response control, results from a recent neurophysiological study with the CPT-task are consistent with this prediction, and indicate that such deficits are indeed particularly pronounced in this comorbid group [21]. Deficits in executive functioning in general, and inhibition deficits in particular are also explained by other neurophysiological theories focusing on either AD/HD or ODD/CD. For ODD/CD [22-24], it has been argued that deficits of the prefrontal cortex leads to reduced orienting and arousal, both of which predispose individuals to stimulation-seeking, disinhibition and attention deficits, and thereby to antisocial behaviour.

The ,Stop-signal paradigm'[25] allows investigating well defined response inhibition processes directly. Generally, the subjects perform a simple or a two choice reaction task. In some of the trials, a Stop-signal follows the go-stimulus at a given delay and requires the inhibition of the ongoing response. The longer the Stop-signal-delay (SSD), the more difficult it becomes to inhibit the response. The „horse race“ model of the Stop-task, which assumes a race between the reaction to the primary task and the reaction to the Stop signal, further allows to estimate the "virtual" reaction time to the Stop-signal (SSRT) as a measure for response inhibition performance [25]. In a meta-analysis of the Stop-task, Oosterlaan et al. [26] reported that behavioural studies showed consistently slower SSRT for children with ADHD, but also for children with CD compared to controls, Comparisons between AD/HD and CD as well as between AD/HD+CD and AD/HD revealed no differences. However, inferences based on performance data only may have limited validity, because differences in covert brain mechanisms may lead to similar overt performance [21,27].

A more direct access to brain functions is provided by non-invasive methods such as functional magnetic resonance imaging (fMRI) [28] or event related potentials (ERP) [29]. Briefly, in the blood-oxygenation-level-dependent (BOLD) fMRI, changes in cerebral blood-flow and metabolism related to neuronal activation are measured with high spatial but low temporal resolution reflecting the underlying hemodynamic process. ERPs are voltage topographies and fluctuations recorded on the scalp which reflect neural activation to an event such as the presentation of a stimulus or a response. A major advantage of the ERP technique is the high temporal resolution in the range of milliseconds which allows to measure brain-electrical correlates of information-processing in realtime. A number of studies therefore used electrophysiological or fMRI measures of response inhibition processes in AD/HD [7,27,30-32].

An ERP-study of Brandeis et al. [27] revealed that in AD/HD children, successful Stops differed from Stop-failures with topographic alterations in a microstate which reflected mainly processing of the go-stimulus, whereas normal controls differed at a slightly later stage of processing with increased global-field-power (GFP, the spatial standard deviation of voltages) in Stop-failures compared to correct Stops. Rubia et al. [30] reported that during their fMRI-study, decreased right-inferior-prefrontal activation in AD/HD occurred solely in the Stop-task, and thus hypothesized the "brake system of the brain" [30] to be located right-prefrontal. Pliszka et al. [7] reported for normal controls a negative wave 200 ms after onset of the Stop-signal (Stop-N200) over right inferior frontal electrodes which was reduced in ADHD-children. For both groups, this N200 after successful inhibitions was positively correlated with inhibition performance whereas correlations for Stop-N200 to Stop-failures were not that clear. Following Kok [33], the N200 to the Stop-signal could either reflect a 'red flag' or a subsequent "(action-) inhibitory process, emanating from structures in the prefrontal cortex" [33]. A second finding was that at right-frontal electrode-sites 250–500 ms post Go-signal-onset the control-group displayed greater positivity to failed than successful Stop-trials whereas in the ADHD-group successful trials did not differ from failed ones. This preparatory activity in failed Stop-trials was more positive in controls than in ADHD patients. Further, Dimoska et al. [32] found, despite worse Go-task- and inhibition-performance in AD/HD compared to controls, different activation-patterns at an early stage of processing the Stop-signal. Again, a decreased N200 to the Stop-signal of successful Stops for AD/HD was found, whereas groups did not differ concerning Stop-N2 of Stop-failures. Following Pliszka et al. [7], the authors argued that this N200 would reflect activation of inhibitory processes. However, in contrast to Pliszka et al. their auditory evoked N200 was generally larger to failed than to successful Stops. Overtoom et al. [31] found slower SSRT and decreased inhibition performance for AD/HD compared to normal controls. Interestingly the study showed no N200-effects to the Stop-signal. This could be due to the use of an auditory Stop-signal, as Falkenstein et al. [34] found a Nogo-N2 which was smaller for auditory compared to visual stimuli despite similar performance in both modalities which could indicate that inhibition is related to a pre-motor level.

There is an ongoing debate whether the Nogo-N200 reflects inhibitory processes per se [33-37], or conflict monitoring [38-40] which may initiate inhibition. We did not intend to distinguish between these two models. Both of them predict that the Stop-N200 is related to inhibition performance: while the inhibition theory relates diminished Stop-N200 amplitudes directly to an impaired central inhibition mechanism, the conflict-signal theory suggests that impaired triggering of the inhibitory mechanisms is responsible.

Taken together, studies strongly suggest difficulties in response inhibition paralleled by neurophysiological deviances for children with AD/HD compared to normal controls, but to our knowledge there is no such evidence for ODD/CD and comorbid AD/HD+ODD/CD. Thus, the aim of this study was threefold, as we intended (1) to replicate the neurophysiological finding of Brandeis et al. [27] and of Pliszka et al. [7] concerning both early pre-Stop-signal processing and the later Stop-N200-differences between controls and children with AD/HD; (2) to clarify whether children with ODD/CD and especially those with comorbid AD/HD+ODD/CD also display an inhibitory-deficit as hypothesized according to Quay's model, i.e. a slower SSRT and slower Stop-failure reaction-times paralleled at the neuronal level by a reduced Stop-N200-amplitude; and (3) we wanted to test whether an additive model of AD/HD and ODD/CD explains response-inhibition performance of children with comorbid AD/HD+ODD/CD.

## Results

### Behavioural data

The groups did not differ in terms of correct Go-reaction-times (F_(3,35) _= 1.97, p > .13), standard deviation of Go-reaction-time (F_(3,35) _= 1.79, p > .17), or accuracy as reflected by percentage of correct Go-trials (F_(3,35) _= 1.43, p > .25, Table 1). A significant partial-correlation between IQ and percentage of correct go-trials was found (r_part _= .45, p < .01). There were also no differences between inhibition-functions (group (F_(3,35) _= 1.60, p > .20) and group*SSD (F_(6,70) _= 1.61, ε = .95, p > .16)).

**Table 1:** Performance Data

		**Group**						
					
		**Controls (N) N = 11**	**AD/HD (A) N = 10**	**AD/HD+ODD/CD (AO) N = 11**	**ODD/CD (O) N = 8**	**ANCOVA (covariate "age")**		
								
**Measure**		**Mean (SD)**	**Mean (SD)**	**Mean (SD)**	**Mean (SD)**	**F**_**(3,35)**_	**p**	**Planned contrasts**
Go-reaction-time (ms)		598 (81.6)	583 (46.2)	594 (52.7)	649 (109.9)	1.97	.14	
SD of Go-reaction- time		161 (43.2)	157.6 (28.4)	155.5 (25.6)	188.5 (49.7)	1.76	.17	
Percentage of correct Go-trials		88.4 (.08)	82.8 (.09)	79.9 (.11)	84.0 (.08)	1.43	.25	
Stop-failure reaction-time (ms)		450 (37.6)	492 (50.5)	502 (50.2)	477 (57.5)	3.70*	.02	N < A*, AO*, O*/A = AO = O
SSRT at 250 ms SSD (ms)		245 (33.9)	272 (47.4)	256 (53.1)	274 (49.8)	3.41*	.03	N < A*, O*/N = AO/AO < A*, O*
Inhibition-function (percentage of Stop failures)	100 ms SSD	3.9 (5.5)	11.0 (9.9)	9.1 (9.9)	5.8 (5.0)	Group: F_(3,35) _= 1.50, p = .21		
	250 ms SSD	30.0 (11.1)	40.3 (16.1)	29.9 (9.6)	27.9 (13.2)			
	700 ms SSD	88.8 (12.0)	89.8 (7.0)	90.8 (6.3)	84.3 (12.5)	Group*SSD: F_(6,70) _= 1.61, ε = .95, p = .16		

* one-tailed, p < .05

However, groups differed in their Stop-failure-reaction-times (F_(3,35) _= 3.70, p = .02) with control children being faster than all clinical groups; no differences were found among the clinical groups. Stop-failure-reaction-time was correlated with IQ (r_part _= .43, p < .01). There were also group-differences in SSRT (F_(3,35) _= 3.41, p > .03) with slower SSRT for the pure AD/HD and ODD/CD groups compared to controls, but not for the comorbid AD/HD+ODD/CD which displayed faster SSRT than AD/HD and ODD/CD. In the 2*2 ANCOVA-design, there were no main effects for AD/HD (F_(1,35) _= .14, p > .71) or ODD/CD (F_(1,35) _= .04, p > .85) on SSRT; but an interaction-effect AD/HD*ODD/CD (F_(1,35) _= 10.21, p < .01).

### Brainmapping

For correct Go-trials, only the second microstate 200–272 ms post go-signal-onset revealed group-differences in GFP (F_(3,35) _= 4.53, p < .01) with lower values for all clinical groups compared to controls (see Table 2). No differences in topography were found (Pillai-Spur F_(12,102) _= 1.15, p > .33).

**Table 2:** Analyses of Microstates

	**Microstate**					
	
	**I**	**II**	**III**	**IV**	**V**	**VI**
						
Correct Go: GFP^a^	1.25	4.53* C>A*, AO*, O*	.88	.88	1.67	
Correct Go: Topography^b^	1.21	1.15	.66	1.29	1.63^+^	
Successful Stop: GFP	.74	4.28* C>A*, AO*, O*	.70	2.33^+ ^C>AO^+^, O*	.51	.83
Successful Stop: Topography	.62	1.06	1.16	1.33	1.18	1.52

* p < .05, for comparisons: one-tailed

^+ ^p < .10, for comparisons: one-tailed

^a ^F_(3,35)_, covariate "age"

^b ^multivariate Pillai's-trace F_(12,102)_, covariate "age"

In successful Stops, groups again differed in the second microstate in GFP (F_(3,35) _= 4.28, p = .01) with higher GFP for controls compared to all clinical groups whereas topography did not differ (Pillai-Spur F_(12,102) _= 1.06, p > .4). The fourth microstate, related to the Stop-N200, revealed only an overall trend towards group-differences in GFP (F_(3,35) _= 2.33, p < .1) with ODD/CD lower than controls; groups did not differ in topography (Pillai-Spur F_(12,102) _= 1.33, p > .2).

### Stop-N200

**Figure 1:**
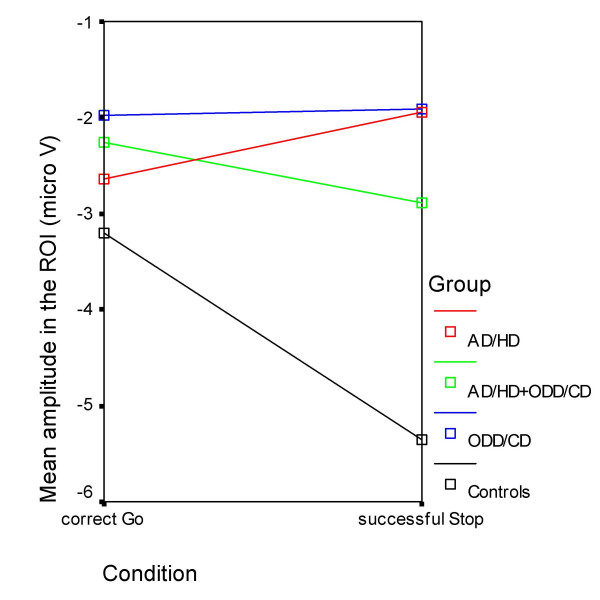
**Mean Amplitudes in the region of interest**. Mean amplitudes in the ROI for correct Go-trials and successful Stops. Normal controls (black), AD/HD (red), AD/HD+ODD/CD (green) and ODD/CD (blue).

**Figure 2:**
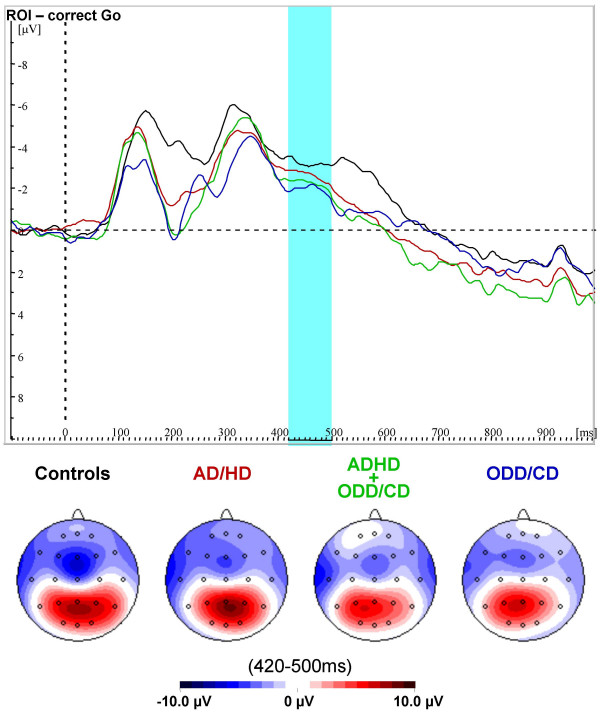
**ERPs for correct Go-trials**. Grand-average waveshapes from the region of interest (F4/F8), and spline-interpolated maps for correct Go-trials for normal controls (black), AD/HD (red), AD/HD+ODD/CD (green) and ODD/CD (blue). There were no group-differences and no negative peaks in the region and time window of interest.

**Figure 3:**
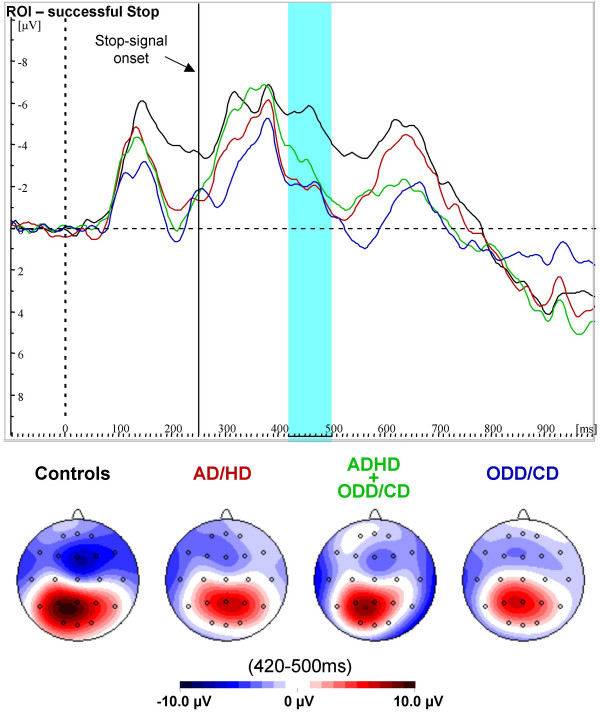
**ERPs for successful Stop-trials**. Grand-average waves in the region of interest and spline-interpolated maps for successful Stop-trials for normal controls (black), AD/HD (red), AD/HD+ODD/CD (green) and ODD/CD (blue). Only normal control children display a negative peak approximately 210 ms after onset of the Stop-signal.

In the frontal region of interest, no main-effect of "condition" (F_(1,35) _= 1.1, p > .3), but a trend for an interaction-effect "condition*group" (F_(3,35) _= 2.5, p = .07) was found at the given time window 170–250 ms post Stop-signal-onset. Separate ANCOVAs for both levels of the "condition"-factor revealed that there were no amplitude differences between the groups for correct Go-trials (F_(3,35) _= .80, p = .50), but significant differences of mean amplitude in ROI for successful Stop-trials (F_(3,35) _= 3.15, p < .04). These differences were reflected by increased negativity in controls compared to all clinical groups which did not differ among themselves (see Table 3 and Figures 1, 2, and 3).

**Table 3:** Electrophysiological Data

	**Group**						
	**Controls (N) N = 11**	**AD/HD (A) N = 10**	**AD/HD+ODD/CD (AO) N = 11**	**ODD/CD (O) N = 8**	**ANCOVA (covariate "age")**		
							
**Measure**	**Mean (SD)**	**Mean (SD)**	**Mean (SD)**	**Mean (SD)**	**F**_**(3,35)**_	**p**	**Planned contrasts**
Go-Trial ROI^a ^mean amplitude (μV)	-3.20 (1.68)	-2.64 (2.10)	-2.25 (2.36)	-1.98 (2.18)	.80	.50	
Stop-Trial ROI^a ^mean amplitude (μV)	-5.36 (1.69)	-1.94 (4.00)	-2.89 (2.21)	-1.90 (4.20)	3.15	.04*	N<A*, AO*, O*/A = AO = O
Stop-N200 ROI^a ^mean amplitude (μV)	-2.16 (1.60)	.69 (3.43)	-.63 (1.76)	.07 (2.02)	2.54	.07^+^	N<A*, AO^+^, O*/A = AO = O

* one-tailed, p < .05

^+ ^one-tailed, p < .10

^a ^Region of interest, mean of electrodes F4 and F8 at 420–500 ms post Go-signal onset

**Figure 4:**
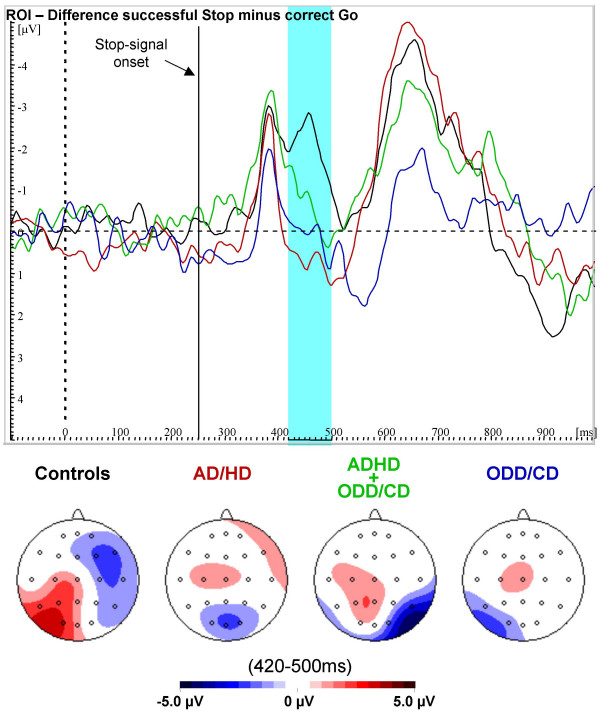
**Difference ERPs (successful Stop minus correct Go)**. Difference waves and spline-interpolated difference maps between event-related potential grand means of successful Stop-correct Go-trials in the region of interest for normal controls (black), AD/HD (red), AD/HD+ODD/CD (green) and ODD/CD (blue). A clear Stop-signal N200 is present only for normal controls.

In order to clarify the interaction "condition*group" which reflects the Stop-N200, planned comparisons of the difference between mean amplitude of successful Stop and correct Go-trials were computed (Figure 4). The (difference-) Stop-N200 was increased for normal controls compared to pure AD/HD and ODD/CD, but there was just a trend for increased negativity in controls compared to comorbid AD/HD+ODD/CD. Again, clinical groups did not differ. The Stop-N200 analysed with the 2*2 ANCOVA revealed no main effects AD/HD or ODD/CD (F_(1,35) _= 2.08, p = .16 and F_(1,35) _= .39, p = .54, respectively) but again an interaction AD/HD*ODD/CD (F_(1,35) _= 4.63, p < .04). For the total sample, this Stop-N200 correlated positively with the speed of the inhibition process (r_part _= .31, p < .05).

## Discussion

The Stop-task was used to investigate inhibitory response control in children with AD/HD, ODD/CD and comorbid AD/HD+ODD/CD in comparison to normal controls. While processing the Go-signal, all clinical groups displayed reduced map strength in a microstate attributable to initial orienting, consistent with previous work [7,27]. A novel finding was that this Go-signal related reduction occurred on both correct Go-trials and successful Stops rather than just on Stop-failures, indicating a more general deficit than reported in previous work. Moreover, these earlier studies had reported a different topography of brain electrical activity with frontal positivity whereas in this work particularly in controls frontal negativity emerged. One explanation may be that participants in our sample showed less Stop-failures than for instance participants of Brandeis et al. [27] did: In this study, percentages of Stop-failures were 30% for controls and 40% for children with AD/HD but 48% and 51% respectively in Brandeis et al. [27]. This could have invoked the same inhibitory or conflict monitoring mechanism as reflected later on by the Stop-N200.

Normal control children displayed a right anterior negativity to the Stop-signal which could reflect response inhibition processes in the right prefrontal cortex [7,32,33], or the mechanism triggering such an inhibitory process. Although deciding between these alternatives is beyond the scope of this study, the right-frontal topography of this Stop-N200 [7] slightly favors the inhibition explanation, and differs from that of the "conflict" Nogo-N200 at fronto-central electrodes [21,39,40].

Not only the Stop-N200 effect, but also its attenuation in AD/HD children as first described by Pliszka et al. [7] could be replicated. It can not be attributed to differences in processing the primary-task at that stage, because group-amplitudes did not differ in this region of interest in Go-trials. Along with this, children with AD/HD performed poorer than normal controls in behavioural response inhibition. Their Stop-signal reaction-times and their reaction-times in Stop-failures were considerably slower than those of normal controls, indicating an even slower inhibitory process, consistent with the majority of previous work [8,9]. Alternative explanations such as 'clumsiness' or 'poor motor control' seems not to be valid since there was a lack of group-differences in other performance measures not related to behavioural inhibition, such as go-reaction-time or accuracy.

The lack of differences between inhibition functions could be due to the adaptive instructions. These were used in this version of the Stop-task to prevent extreme speed-accuracy-tradeoffs at the fixed medium stop signal delay. Such fixed Stop signal delays are advantageous for ERP studies, but suboptimal for deriving inhibition functions [41]. Still, we note that it is crucial for any Stop-task to balance between the strategies avoiding every Stop-failure or responding so fast that no stopping is possible either implicitly (with standard instructions like "respond as quickly and as accurately as possible to the primary stimulus, as well as to inhibit the response on the appearance of the Stop signal" [8]) or explicitly as is done here. The widely reported finding that children with AD/HD display more variable reaction-times could not be replicated here, maybe also because of the adaptive instructions.

Inhibition deficits were not limited to children with AD/HD, but also characterized children with ODD/CD, as predicted by the model of Quay. Their Stop-N200 was also reduced compared to normal controls, and did not differ from AD/HD and comorbid AD/HD+ODD/CD. The latter finding extends the commonality between AD/HD and ODD/CD to the neurophysiological level, which is in contrast to Quay's theory of conduct disorder postulating an intact behavioural inhibition system.

Surprisingly, children with comorbid AD/HD+ODD/CD tended to be somewhat less impaired than the other clinical groups. Their inhibition process (as reflected by SSRT) was not significantly slower than in normal controls, and was even faster than in the other clinical groups. However their Stop-failure reaction-times were slower compared to normal controls and similar to that of the other clinical groups. Hence, inhibition performance was by no means most impaired in comorbid AD/HD+ODD/CD. Although there was only a trend for decreased Stop-N200 mean amplitude compared to normal controls, no differences were found compared to the pure groups, which again stands in contrast to Quay's theory.

Consistent with this pattern, the 2*2 ANCOVA with between subject factors "AD/HD" and "ODD/CD" revealed no main- but strong interaction-effects for the most important measures of inhibition, indicating that effects of AD/HD and ODD/CD symptoms on response inhibition were not additive but sub-additive. This supports the conclusion of Banaschewski et al. using the CPT [42] who argue against the view that comorbid AD/HD+ODD/CD is a hybrid or a phenocopy of AD/HD or ODD/CD. The present results suggest that this conclusion is not task specific.

However, since CPT and Stop-task were performed by partly the same sample of children, an independent replication with a larger sample size is needed to further support this view.

Although some evidence for inhibition deficits in AD/HD has been obtained using the CPT [43,44], it was also found that neither commission errors nor the Nogo-N200 enhancement had differed between the groups in the cued CPT [21,44]. We note that there are clear differences in what has to be inhibited in these two tasks: In the CPT, participants have to withhold a prepared but not yet initiated response and made only a few false alarms. In the Stop-task, participants have to stop an already ongoing response which often failed. These two types of inhibition have to be differentiated (see e.g., Barkley [2,3]).

## Conclusion

While all clinical groups displayed similarly attenuated neural signs of go-signal processing, the subsequent response inhibition deficits further separated the clinical groups. Both children with AD/HD and ODD/CD-patients were found to be impaired in behavioural response inhibition. Also, both groups displayed reduced neuronal inhibition as reflected by smaller right-frontal Stop-N200 amplitudes; for AD/HD this is in agreement with Quay's model of psychopathology whereas for ODD/CD predictions of that model were violated. Hence, the inhibition-deficit concerning "stopping of an ongoing response" is by no means specific for AD/HD. In addition, the comorbid group with AD/HD+ODD/CD which should display the most severe deficits was found to be even somewhat less impaired than the "pure" groups, indicating that the comorbid condition may represent a separate disorder distinct from AD/HD and ODD/CD.

## Limitations

The study is limited by its small sample size and by the fact, that another attention test was administered beforehand. Valid performance data concerning inhibition performance was only available for one fixed SSD. Since SSRT is only estimated from one fixed SSD with less than the optimal Stop-failure rate of 50% [41], its reliability and its sensitivity to group-differences is decreased compared to other strategies to estimate SSRT.

## Methods

### Subjects

Fifty-eight boys aged 8–14 years participated in the study on the basis of informed consent by child and parent with approval of the local ethics committee; all had normal or corrected to normal vision, a full scale IQ above 80 and understood the Stop-task-instructions. Some datasets were deleted a priori because of more than 20% omissions of go-trials (for 2 controls, 1 AD/HD, 1 AD/HD+ODD/CD and 2 ODD/CD), and some were lost due to artifacts in the EEG (1 control, 1 AD/HD, 3 AD/HD+ODD/CD and 1 ODD/CD) or due to age-matching the groups.

Datasets of a total of forty participants were thus analysed. They belonged to one of three clinical subgroups with the ICD-10 diagnoses hyperkinetic disorder (F90.0, N = 11), oppositional defiant/conduct disorder (F91, F92, N = 8), hyperkinetic conduct disorder (F90.1 N = 11) or to a group of 11 healthy controls (Table 4).

**Table 4:** Sample description

	**Group**						
				
	**Controls (N) N = 11**	**AD/HD (A) N = 10**	**AD/HD+ODD/CD (AO) N = 11**	**ODD/CD (O) N = 8**	**ANOVA**		
							
**Measure**	Mean (SD)	Mean (SD)	Mean (SD)	Mean (SD)	**F**_3;36_	p <	**Scheffé-Tests **^**a**^
Full-scale-IQ	110,7 (15,1)	94,4 (6,9)	93,0 (9,4)	96,9 (10,5)	5,9	0,01	N > A, AO
Age (in months)	130,8 (18,9)	130,1 (18,0)	123,7 (18,5)	131,5 (27,4)	0,3	0,81	
CBCL^b^							
							
Social withdrawal^b^	52,0 (4,6)	59,8 (6,5)	58,7 (7,7)	63,9 (9,9)	4,5	0,01	N < O
Somatic complaints^b^	56,3 (5,0)	56,2 (7,9)	57,2 (9,6)	57,4 (6,1)	0,1	0,98	
Anxiety/Depression^b^	50,3 (0,9)	57,2 (7,1)	64,3 (12,0)	67,3 (8,8)	8,4	0,01	N < O, AO
Social problems^b^	50.0 (0.0)	62.0 (8,7)	58,2 (8,2)	62,3 (11,5)	5,3	0,01	N < A, O
Thought problems^b^	50,6 (2,1)	54,2 (4,9)	61,4 (9,4)	61,8 (10,1)	5,9	0,01	N < O, AO
Attention problems^b^	50,3 (0,5)	67,4 (6,6)	67,3 (6,9)	69,1 (6,5)	25,5	0,01	N < A, AO, O
Delinquent behaviour^b^	50,8 (2,7)	59,7 (8,7)	71,6 (10,2)	69,9 (10,0)	14,1	0,01	N < AO, O/A < AO
Aggressive behaviour^b^	50,3 (0,9)	63,7 (8,7)	78,6 (12,0)	75,8 (14,6)	17,4	0,01	N < A, AO, O/A < AO
Internalizing symptoms^b^	44,3 (7,6)	57,4 (10,0)	62,3 (11,2)	65,8 (8,7)	10,0	0,01	N < A, AO, O
Externalizing symptoms^b^	38,3 (7,7)	63,1 (7,6)	75,3 (9,1)	73,3 (9,8)	41,9	0,01	N < A, AO, O /A < AO

^a ^α < 0,1

^b ^Child Behaviour Checklist, T-scores

Children of the clinical groups were sequential referrals to the Department of Child and Adolescent Psychiatry of the University of Göttingen who met no other psychiatric diagnoses except reading and/or spelling disorders (N = 15), enuresis (N = 1) or encopresis (N = 1). The diagnosis of a hyperkinetic disorder was concordant with the DSM-IV diagnosis of ADHD-combined type. Control children met no other psychiatric diagnoses than reading and/or spelling disorders (N = 4). Diagnoses were verified by senior board-certified child psychiatrists. All children underwent standardized IQ-testing with the German versions of the WISC-R [45] or Culture Fair Intelligence Test (CFT [46]). The CFT was used only in 5 cases (for 3 controls, 1 ADHD and 1 ADHD+ODD/CD).

Groups were matched by age but not by IQ, with lower IQs for the psychopathological groups compared to normal controls (F(3/36) = 5.9, p = 0.01).

One-way analyses of variance (ANOVAs) were carried out to explore group-differences concerning the scales of the parent-rated Child Behaviour Checklist (CBCL [47]). There were group differences for all CBCL-scales except somatic complaints (F(3/36>4.5, p < 0.01), results of post-hoc Scheffé-Test are shown in Table 4.

### Stimuli and task

The Stop-task consisted of eight blocks with 40 trials each and was identical to that used by Brandeis et al. [27] and Rubia et al [8]. Stimuli were presented in the central 2*2 cm square of a VGA monitor at 120 cm viewing distance with fixation marks above and below the scene. Each trial started with the presentation of an aeroplane in side view, suggesting that is was 'flying to' the left or to the right, and the children had to press a button corresponding to the planes flying direction with the index finger of their left or right hand. They were also told that sometimes a "little man" with his hands raised would follow, indicating that they should withhold their response. This should be easy when the "little man" occurred early, but they should no longer be able to stop their prepared response when the man was late.

Altogether, the "little man" Stop-signal occurred in 50% of the trials. The three fixed Stop-signal-delays (SSD) were 100 ms (10% of all trials), 250 ms (30%) or 700 ms (10%). The summed duration of the two signals was in every case 800 ms (800+0 ms, 100+700 ms, 250+550 ms, 700+100 ms) and a trial was presented every 1650 ms.

Identical instructions were given to all groups before the practice-block, and were repeated after a block in case the child made more than 25% Stop-failures in the short SSD or less than 75% Stop-failures in the long SSD condition. Thus the short and long SSDs aiming at 0% and 100% stop failures provided a time frame within which the child's response should occur, therefore only the medium SSD was analysed. If there were less than 33% or more than 66% correct Stops at the medium SSD in a given block, additional instructions were given to slow down or speed up responses, respectively. These adaptive instructions prevent undesired strategies in performing the task, such as extreme speed-accuracy-tradeoffs yielding very frequent or very rare Stops at the fixed medium SSD [27]. The inhibitory deficits detected in ADHD children are comparable when using this Stop task with fixed SSDs, or the standard version with adaptive SSDs [8].

### ERP recording and processing

An ERP was recorded using a Neuroscan recording system with calibrated technical zero baselines and Nihon Kohden Ag/AgCl electrodes attached to the skin with Grass EC-2 electrode-cream. Sampling-rate was 250 Hz and cut-off frequencies were 0.1 and 50 Hz on all 10–20 electrode positions using FCz as recording reference and a ground electrode placed on the forehead. Vertical and horizontal electro-occulograms (EOGs) were recorded simultaneously from electrodes above and below the left eye and at the outer canthi. Impedances were kept below 5 kΩ, further analyses were computed with the Vision Analyzer 1.05 software.

The EEG was transformed to the average reference of the 10–20 electrodes plus Fpz and Oz. Data were filtered offline (Butterworth, 0.1 to 30 Hz, 24 dB/oct.). For eye movement correction the method of Gratton & Coles [48] without raw average subtraction was used. Trials with performance errors (side-errors, failed Stops and Go-reaction-times faster than 200 ms), amplifier saturation or artefacts exceeding +-200 μV amplitude or more than 200 μV amplitude difference in a segment -100 ms to 1500 ms around go-signal-onset were rejected; remaining segments were subsequently checked visually. A 100 ms pre-stimulus baseline (referred to the go-signal-onset) was taken as zero. Averages for successful Stops in the medium SSD contain at least 25 sweeps, correct Go-Averages contain at least 90 sweeps. Groups did not differ in both numbers of accepted sweeps.

### Analyses

#### SSRT

Reaction-times shorter than 200 ms and Go-Trials with side-errors were excluded from all analyses. SSRT was estimated only for the medium SSD because there were too few Stop-failures in Stop-trials with short and too many in long SSD. The classic approach to calculate SSRT for a specific SSD is to rank-order reaction-times of the go-trials, multiply the probability for a Stop-failure with the number of go reaction-times which yields n, take the go reaction-time of the n*th *rank and subtract the SSD [9,49]. This leads to certain difficulties: for instance, if a participant makes no Stop-failure in the questioned SSD; the probability for a Stop-failure will be zero and there is no zero-rank of go reaction-times. But this participant has initiated a quite well-working Stop-process with which our applied theory can not cope. On the other hand, if a participant was not able to Stop even once, the algorithm would yield a wrong estimate of SSRT. Taken together, the algorithm stated above could lead to an undefined state and to wrong results which makes it susceptible of formal refutation.

Hence, we used a slightly different strategy: We took the probability for a Stop-failure, multiplied it with the total number of correct go-reactions and truncated the result. There we got the rank n (if there is any) of the Go-reaction-time which was just too fast to be stopped, the (n+1)-rank (again, if there is any) denotes the fastest Go-reaction-time slow enough not to yield a Stop-failure, and the mean of the two minus their Stop-signal-delay would yield a good estimate for SSRT. This brings into account, that the distribution of Go-reaction-times is discrete rather than continous.

Applied to a dataset without Stop-failures, we can only determine one border of the area of reaction times in which correct Stops and Stop-failures occur; we only know reaction-times which are slow enough not to evoke a Stop-failure. The best we can say therefore is that the SSRT shall be faster than the fastest Go-reaction-time with Stop-signal-delay subtracted. If a dataset contains no correct Stops, we only know that every Go reaction-time was too fast to be stopped, but we do not know anything more; simply taking the fastest Go-reaction-time with SSD subtracted as SSRT would be wrong. Because of this indeterminacy of Stop-signal-reaction-time, participants with no Stop-failures as well as participants with no correct Stops need to be excluded from analyses. This was not necessary for the dataset presented.

### Brain-mapping

Microstates were determined on the total group's grand mean. Borders were set at times with minimal global-field-power (GFP) indicating low map-strength, plus maximal dissimilarity (Diss, the GFP of the difference between successive normalized maps) reflecting high topographic instability [27,50]. In contras, components extracted by principal component analysis (PCA) were only statistically defined as sources of variance and may not necessarily be grounded by physiological components [51,52].

**Figure 5:**
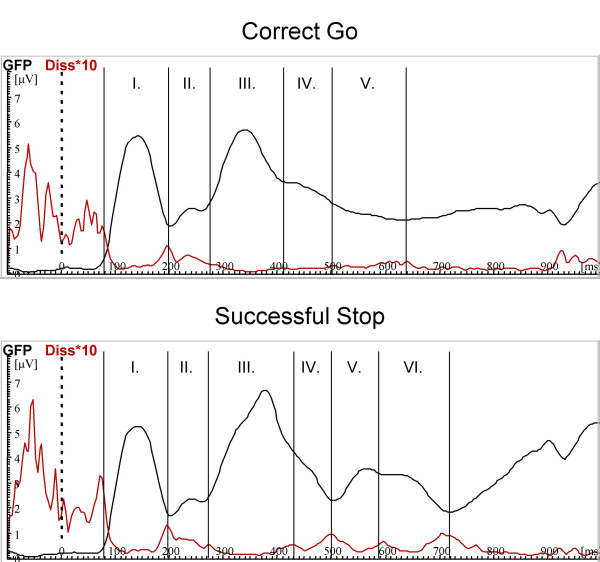
**Microstate estimation according to GFP and Diss**. Adaptive segmentation of the total groups grand mean from correct Go (top) and successful Stop (bottom). Microstate boarders were determined by relative minima of GFP (black) together with relative maxima in Diss (red, for better scaling multiplicated with 10) Correct Go-trials revealed five microstates (76–196 ms, 200–272 ms, 276–412 ms, 416–504 ms, 508–640 ms), successful Stops six microstates (76–196 ms, 200–272 ms, 276–428 ms, 432–504 ms, 508–592 ms, 596–724 ms).

**Figure 6:**
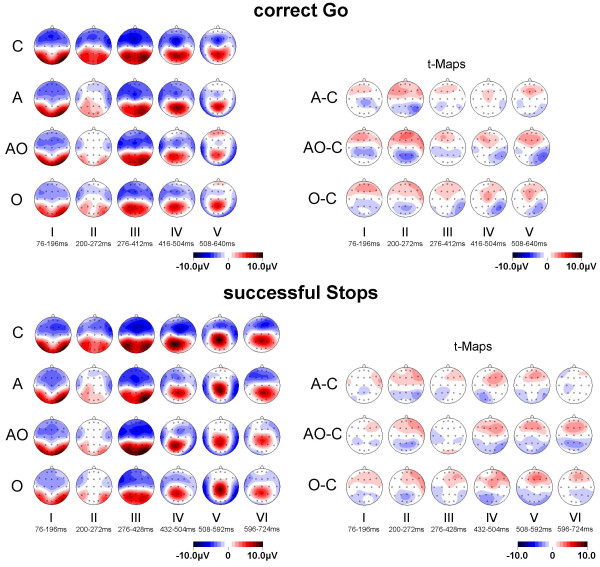
**Microstate-maps and t-maps for correct Go and successful Stop-trials**. Spline-interpolated microstate-maps for normal controls (C), children with AD/HD (A), ODD/CD (O) and AD/HD+ODD/CD (AO) and additional exploratory t-maps with comparisons of clinical groups vs. controls. Unadjusted two-tailed significance-level is reached at t_(17 to 21) _> 1.7 p < .05.

For correct Go, five microstates were found (76–196 ms, 200–272 ms, 276–412 ms, 416–504 ms, 508–640 ms), correct Stops revealed six (76–196 ms, 200–272 ms, 276–428 ms, 432–504 ms, 508–592 ms, 596–724 ms, see Figure 5). For each microstate a mean map with its GFP and summary measures of topography (centroids) [50] were computed (Figure 6).

### Stop-N200

In this version of the Stop-task, processing of the Stop-signal is fully time-locked with the preceding go-stimulus and thus highly confounded with go-signal processing. Because of this, differences between features of Stop- vs. Go-trials were analysed in a repeated measure-design; it is likely that such differences were caused by processing the additional Stop-signal on Stop- trials. Separate analyses of the conditions and inspection of the segment *t*-maps of the group differences in the raw conditions were used to exclude alternative interpretations. The term "Stop-N200" as used here thus refers to this difference between mean negativity in the ROI of successful Stop and correct go-trials.

Visual inspection of the normal controls' grand mean of correct Stops revealed a negativity peaking at about 460 ms after go-signal-onset (or 210 ms after Stop-signal-onset) at right-frontal electrodes which was absent in go-trials. For further analyses, the mean amplitude in a time-window 420–500 ms after onset of the Go-signal was computed separately for correct go- and successful Stop-trials. The time-window used to study this local effect is almost identical with microstate IV found with the Brainmapping-approach.

In order to localize the region of interest in this time-window for the sub-sample of normal controls a repeated-measure-ANCOVA with within-subject-factors "condition" (successful Stop vs. correct Go) and electrode-sites "anterior-posterior" (3 levels) and "left-right" (5 levels) was computed for the vector-length-normalized dataset. Vector-normalization is necessary, because "condition*location" interactions can result from multiplicative changes in source strength between conditions without specific differences concerning locations [53]. The ANOVA revealed a significant interaction-effect "condition*left-right" (F_(4,80) _= 5.93, ε = .542, p = .01) and an interaction "condition*left-right*anterior-posterior" (F_(8,80) _= 3.00, ε = .424, p = .04). Exploratory analyses of repeated measure "condition" for each electrode separately (without vector normalization) revealed significant differences between conditions only at electrodes F4 (F_(1,10) _= 15.1, p = .003) and F8 (F_(1,10) _= 15.2, p = .003) with increased negativity in trials with successful Stops as well as increased positivity at P3 (F_(1,10) _= 20.9, p = .001). Therefore the mean-amplitude of the adjacent right-anterior electrodes F4 and F8 in this time-window were used as region of interest (ROI) in order to analyse Stop-signal-N200, similar to Pliszka et al. [7].

To test whether dependent measures were confounded with developmental effects (the higher age, the higher performance and the lower ERP-amplitudes), simple correlations with "age" were computed across all groups. For Go-reaction-time (r = -.40*), Stop-failure reaction-time (r = -.72*), SSRT (r = -.76*) and mean amplitude in the ROI for correct Go (r = .27*) and successful Stop (r = .27*) developmental effects occurred, whereas the N200 of the difference-wave in the ROI between correct Go and successful Stop was not affected by age (r = .13; all one-tailed tested, * p < .05).

Therefore, age was taken as a covariate for all comparisons to reduce error-variance due to developmental effects and thus increase statistical power.

### Statistical tests

Go-reaction-time, Stop-failure reaction-time and SSRT were analysed with one-way analyses of covariance (ANCOVAs) with between-subject-factor "group" and covariate "age". In case of overall-differences between groups, planned contrasts were computed in order to test the hypothesis that clinical groups display decreased performance (slower SSRT, Go- and Stop-failure-reaction-time) compared to normal controls. The inhibition-function of probabilities of Stop-failures for each SSD was analysed with a two-way repeated measure ANCOVA with within-subject-factor "SSD", between-subject-factor "group" and covariate "age".

All microstates of correct Go and successful Stop were analysed exploratory concerning GFP with one-way ANCOVAs with between-subject-factor "group" and covariate "age". Differences in topography as reflected by locations of centroids were analyzed with MANCOVAs of dependent variables "location of positive and negative centroids" (left to right and anterior to posterior for each), covariate "age" and between-subject-factor "group" [54].

Group-comparisons of Stop-signal-N200 were analysed with a two-way repeated-measure ANCOVA with within-subject-factor "condition" (correct Go vs. successful Stop), between-subject-factor "group" and covariate "age". In case of significant differences, further one-way ANCOVAs and additional planned contrasts were computed. In order to correct results of repeated-measure ANCOVAs from violations from sphericity, Greenhouse-Geisser ε and p-values for corrected degrees of freedom were reported.

To test an additive model of effects on response inhibition, separate 2*2 ANCOVAS with between-subject factors "AD/HD" and "ODD/CD" and covariate "age" were computed for the main dependent variables SSRT and Stop-N200.

Because of small sample size, even trends with p < .10 will be reported for hypothesized group and condition differences. Cohen's standardized mean difference for the sample with age taken as covariate d_sc _were computed (Table 5).

**Table 5:** Effect size (Cohen's d) for the main dependent variables

		**Controls (N)**	**AD/HD (A)**	**AD/HD+ODD/CD (AO)**	**ODD/CD (O)**
					
**Measure**		**effect-size d**_**sc **_^**a**^	**effect-size d**_**sc **_^**a**^	**effect-size d**_**sc **_^**a**^	**effect-size d**_**sc **_^**a**^
Stop-failure reaction-time (ms)	**A**	-1.26*			
	**AO**	-1.22*	.03		
	**O**	-.87*	.39	.36	
SSRT at 250 ms SSD (ms)	**A**	-.91*			
	**AO**	.06	.96*		
	**O**	-1.08*	-.18	-1.13*	
Go-Trial ROI^b ^mean amplitude (μV)	**A**	-.29			
	**AO**	-.57	-.28		
	**O**	-.60	-.31	-.02	
Stop-Trial ROI^b ^mean amplitude (μV)	**A**	-1.16*			
	**AO**	-.93*	.22		
	**O**	-1.15*	.01	-.20	
Stop-N200 ROI^b ^mean amplitude (μV)	**A**	-1.15*			
	**AO**	-.66	.49		
	**O**	-.89+	.26	-.22	

^a ^d_sc _is the standardized mean difference for the sample, based on the ANCOVA model with age as covariate. This is done in order to account for developmental effects.

^b^ Region of interest, mean of electrodes F4 and F8 at 420-500 ms post Go-signal onset

* one-tailed, P < 0.05

+ one-tailed, P < 0.10

Since groups were not IQ-matched, influences of IQ on dependent measures were analysed using partial correlation coefficients with covariate "age" and will be reported in case of significance.

### Procedure

The psychophysiological experiment took place in a video-controlled, noise-protected and slightly dimmed room at the Department of Child and Adolescent Psychiatry at the University of Göttingen. Participants sat in a dentist-chair during electrode-attachment and task performance. Throughout the tasks, they could communicate with the experimenter via intercom. At first, a CPT lasting at about 11 minutes was performed [21,42], followed by this Stop-Task of approximately 10 minutes duration.

## Competing interests

The author(s) declare that they have no competing interests.

## Authors' contributions

BA performed analyses and drafted the manuscript, TB, DB, HH and AR conceived the study and helped to draft the manuscript.
